# Discrimination and well-being amongst the homeless: the role of multiple group membership

**DOI:** 10.3389/fpsyg.2015.00739

**Published:** 2015-06-01

**Authors:** Melissa Johnstone, Jolanda Jetten, Genevieve A. Dingle, Cameron Parsell, Zoe C. Walter

**Affiliations:** ^1^School of Psychology, The University of Queensland, St Lucia, QLDAustralia; ^2^Institute for Social Science Research, The University of Queensland, St Lucia, QLDAustralia

**Keywords:** homeless, discrimination, multiple group membership, well-being

## Abstract

The homeless are a vulnerable population in many respects. Those experiencing homelessness not only experience personal and economic hardship they also frequently face discrimination and exclusion because of their housing status. Although past research has shown that identifying with multiple groups can buffer against the negative consequences of discrimination on well-being, it remains to be seen whether such strategies protect well-being of people who are homeless. We investigate this issue in a longitudinal study of 119 individuals who were homeless. The results showed that perceived group-based discrimination at T1 was associated with fewer group memberships, and lower subsequent well-being at T2. There was no relationship between personal discrimination at T1 on multiple group memberships at T2. The findings suggest that the experience of group-based discrimination may hinder connecting with groups in the broader social world — groups that could potentially protect the individual against the negative impact of homelessness and discrimination.

## Introduction

A large body of work demonstrates that people who are homeless also experience disproportionate rates of health problems and associated social disadvantages (Rosenthal et al., 2006; Echenberg and Jensen, 2009; Scutella et al., 2012). Pervasive discrimination experienced by people who are homeless, particularly discrimination based on access to accommodation and goods and services, contributes to the high rates of poor health (Phelan et al., 1997; Lynch and Stagoll, 2002). Moreover, the discrimination that homeless individuals face is perceived as legitimate (Fiske et al., 2002), not only by the general public, but also by individuals who experience homelessness themselves.

Even though previous research has shown that turning to others may alleviate the negative effects of discrimination on well-being (Branscombe et al., 1999), and that identifying with multiple groups in particular has beneficial well-being effects (Iyer et al., 2009; Ysseldyk et al., 2013; Haslam et al., 2014), it remains to be seen whether these effects will be observed among individuals who are homeless. There are reasons to believe that, given the highly stigmatized nature of homelessness, individuals who are homeless may have limited opportunities to join groups that may protect their well-being when facing discrimination. We examine this prediction in a longitudinal study among individuals who reside at homeless shelters. Before outlining our study, we first elaborate the rationale underlying our prediction.

### Stigma and Discrimination Amongst People Who are Homeless

A large body of work examining a broad range of disadvantaged groups demonstrates that discrimination negatively affects well-being (Kidd, 2007; for a meta-analytic review see Williams et al., 2003; Schmitt et al., 2014). This work identified a number of factors that influence the relationship between perceived discrimination and well-being. Three of these factors are particularly likely to amplify the negative effects of discrimination on well-being for the current sample. We outline these as background to understanding the reasons as to why people experiencing homelessness might face discrimination, and how perceptions of discrimination and the reasons underlying discrimination may affect outcomes.

First, there is evidence that when the stigmatized identity is viewed as to some extent controllable (such as unemployment, drug addiction, or obesity), group-based discrimination has a more harmful effect on well-being than discrimination directed against those with an uncontrollable stigma (such as race or gender). Indeed, negative group-based treatment is more likely to be perceived as legitimate by both the individuals and the perpetrators if directed at people with controllable stigmas compared to uncontrollable stigmas (Weiner et al., 1988; Rodin et al., 1989). Because housing status is perceived as somewhat under an individual’s control, whereby the homeless are often considered to be responsible for their lack of adequate housing (Parsell and Parsell, 2012), homeless individuals are likely to face highly legitimized forms of discrimination, amplifying negative well-being consequences.

Second, despite the fact that individuals who are homeless are perceived as struggling and in need of care and compassion (Kidd, 2004; Benbow et al., 2011; Shier et al., 2011), there is also evidence that homeless individuals are not perceived as fully human (Harris and Fiske, 2006). Research has shown that homeless people as a group are seen as neither competent nor warm, and thus form “the lowest of the low” (Fiske et al., 2002). This elicits the worst kind of prejudice – disgust and contempt – and can make people functionally equivalent to objects (Harris and Fiske, 2006). This further enhances the perceived legitimacy of negative treatment against the homeless and, in turn, further compromises an individual’s ability to cope with discrimination.

Third, people who are homeless are often not only discriminated against because of their housing status, but also face discrimination for other reasons. In particular, these individuals also commonly experience mental illness and/or drug addiction, conditions which are subject to high levels of stigma in society (Barry et al., 2014).

In sum, because homeless individuals face discrimination that is perceived as legitimate, and targeting them for many different reasons, we predict that these individuals’ well-being will be negatively affected. Consistent with this, both qualitative and quantitative work describes the negative impact of discrimination for the homeless on their well-being (Phelan et al., 1997; Lynch and Stagoll, 2002; Kidd, 2007) and homeless individuals describe the experience of discrimination as making the transition out of homelessness and into employment and stable housing significantly more complex and challenging (Milburn et al., 2006; Piat et al., 2014).

### Coping with Discrimination by Turning to Groups

Given the negative relationship between discrimination and well-being, the question presents itself whether there are factors that attenuate the strength of this relationship. Researchers working from the social identity approach (consisting of social identity theory, Tajfel and Turner, 1979, and self-categorization theory, Turner et al., 1987) have shown that individuals often react to discrimination with increased group identification and cohesion, and this can alleviate some of the negative effects on well-being (Branscombe et al., 1999). Known as the rejection-identification model, these effects have been demonstrated amongst historically disadvantaged groups, such as African Americans, women and more recently, international students, and people seeking out body-piercings (Branscombe et al., 1999; Jetten et al., 2001; Schmitt et al., 2002, 2003). According to this model, identification follows rejection (i.e., group-based discrimination) because group membership becomes highly salient when individuals face group-based discrimination. This enhances the distinction between ‘us’ (the stigmatized group) and ‘them’ (the majority group), and strengthens identification with the stigmatized group. In turn, enhanced identification with the stigmatized group counteracts some of the negative consequences of facing discrimination and rejection and protects well-being. From this reasoning, it becomes clear that identification can be a psychological resource that group members can fall back on when facing stressors such as discrimination or rejection (Branscombe et al., 1999).

However, in many ways, the homeless are different to other groups experiencing discrimination for at least two reasons (e.g., women, Asians, African–Americans). First, prior research with people who are homeless suggests that individuals do not necessarily identify with other homeless people or think of themselves as similar to others who are homeless (Parsell, 2010; Walter et al., under review). Indeed, Gowan (2009) demonstrates how people living on the streets actively construct a self-identity and convey a public image as entrepreneurs through routine recycling work that they present as socially valuable. The recyclers deliberately identified themselves as workers as a point to contrast themselves with other homeless people. Second, the ‘group’ homeless people is quite diverse consisting of people of different ages, reasons for being homeless, and opportunities for exiting homelessness. As a result of this diversity, the category homelessness becomes less relevant and meaningful as a framework to organize the experiences of individuals facing homelessness. Both the meaningfulness of the homelessness label to describe the self and the diversity of experience among those categorized as homeless suggests that it may not be meaningful to examine the extent to which identification with others that are homeless affects well-being.

Even though experiencing discrimination might not enhance identification with others who are homeless, it may nevertheless lead homeless individuals to turn to groups for identity–based social support. In a development of the rejection-identification model, recent work has shown more generally that identification with groups (other than those that are targets of discrimination) and joining new groups is associated with better well-being for those facing life stressors. For instance, among those with acquired brain injury, gaining the identity of being “a survivor of brain injury” and having a greater number of social relationships since injury was associated with heightened life satisfaction (Jones et al., 2011). In a similar vein, Haslam et al. (2008) found that, after a stroke, those individuals who were able to maintain membership in multiple groups reported greater well-being. More generally, the benefits of multiple group memberships on well-being is supported by a mounting body of evidence linking multiple group identification and enhanced well-being (Iyer et al., 2009; Ysseldyk et al., 2013; Haslam et al., 2014, see also Thoits, 1983; Biswas-Diener and Diener, 2006).

There are a number of reasons why multiple group memberships offer a ‘social cure’ (Jetten et al., 2012). First group memberships can be seen as psychological resources and if individuals identify with groups, mere membership in such groups protects well-being. If groups are resources, it follows that the more resources an individual has, the better protected they are (Jetten et al., 2014). Second, the more groups that individuals belong to, the more “eggs they have in their basket” to deal with life stressors (Putnam, 2000; Roccas, 2003; Jetten et al., 2009). This provides greater flexibility to deal with stressors in the sense that it enhances the likelihood that one can turn to a suitable group when facing a particular stressor.

### Barriers Toward Maintaining Membership and Joining Groups

Even though the extent to which people who are homeless turn to other groups when they face discrimination might be a good predictor of their well-being, discrimination is likely to be an important barrier to joining new groups. Facing legitimate discrimination, being blamed for their homelessness status (Phelan et al., 1997; Milburn et al., 2006), and internalization of this blame will exacerbate the negative effects of stigma among the homeless. Consistent with this, research has found that self-blame and guilt due to homelessness were the most strongly related to low self-esteem, loneliness, feeling trapped, and suicidal ideation, even beyond the effects of stigma (Kidd, 2007).

Facing discrimination may not only stand in the way of seeking out others to cope with discrimination, those who attempt to draw social support may not be successful and they may encounter further rejection. Specifically, others may not be accepting of those who have been or still are homeless and — because discrimination against the homeless is highly legitimized — might exclude those who want to join their groups or social networks. Consistent with this, it has been found that when members of the disadvantaged group perceive the discrimination they face as legitimate (compared to illegitimate), it will lower identification with others suffering from similar negative treatment and reduce intentions to engage in collective action to address the discriminatory treatment (Jetten et al., 2011, 2013). In sum, we predict that given the pervasiveness and legitimacy of discrimination that people who are homeless face, it might be hard for them to join new groups or to maintain membership in their current groups and this may have negative well-being outcomes.

### The Current Research

To recap, it is not surprising that there are negative consequences associated with discrimination. Building on previous work showing that belonging to groups can act as a coping resource averting some of the negative psychological effects of homelessness, we predict that joining new social groups and/or belonging to multiple groups enhances well-being. However, individuals facing homelessness are different from other minority groups facing discrimination. For instance, they are subject to discrimination from their own friends and family, as well as the mainstream, and are often blamed for being in their predicament.

The aim of this research is to investigate how the experience of discrimination amongst the homeless affects social connections, and subsequent well-being. We explored two forms of discrimination: discrimination that one faces as an individual and discrimination that is due to belonging to a stigmatized group. In line with Jetten et al. (2013), we predicted that in particular perceived *group-based* discrimination would be a powerful predictor of an individual’s ability and motivation to turn to groups. This is because group-based discrimination enhances the salience of the intergroup context and enhances ‘us’ versus ‘them’ perceptions in a way that perceived personal discrimination does not. It is therefore mostly in the former, and not the latter form of discrimination that we would expect that individuals would, ordinarily, be motivated to turn to others with whom they share identity — other groups that are part of a large and inclusive ‘we.’ However, because people who are homeless do not generally identify with others who are homeless and because the discrimination experienced by the homeless is pervasive and seen as legitimate, we predict that group-based discrimination would make it more difficult to join new groups, and even more so than perceived personal discrimination.

We analyzed data from two time points from individuals who were living in homeless shelters at Time 1, controlling for initial levels of well-being. It was expected that the experience of greater degrees of discrimination while in the shelter (Time 1) would stand in the way of developing multiple group memberships (either by joining new groups or by nurturing and expanding existing social relationships) at Time 2. This would be associated with lower levels of well-being at Time 2.

## Materials and Methods

### Participants

Participants were individuals who were residing in one of six homelessness accommodation services run by a charitable organization (The Salvation Army in South–East Queensland, Australia). The Salvation Army is a well-known charity that offers a wide range of services, including accommodation and related support for individuals who are homeless. Nationally, the Salvation Army provides crisis accommodation for over 1000 people per night, with a further 6000 persons housed in non-crisis accommodation.

A total of 119 participants completed an interview and questionnaire at Time 1 (T1 for short), including 56 men and 63 women, with an average age of 35.39 years (range: 19–59; SD = 9.34). At T1, the average time participants had been in the homeless accommodation was 7.5 weeks. Although there is a maximum stay in temporary accommodation with the Salvation Army of 3 months, ‘duration of need’ clauses may be placed on the time limit (i.e., if people need to stay longer, they often can). The Time 2 data (T2, *n* = 76) were collected from participants 2–4 weeks after leaving the service, or 3 months after T1 if they had not yet exited the service.

Participants completed a second interview and questionnaire at T2. Attrition analyses revealed that participants who completed T2 were not significantly different from those who dropped out of the study in terms of gender, age, employment status (at T1), initial levels of alcohol consumption or well-being^1^. Of participants providing data at the second time point, 50% were in stable or supported accommodation at T2. At T1, 18.5% of participants were in some form of paid employment, and 87% received some sort of government benefits, compared with 26% and 80.5% at T2.

### Measures

#### Perceived Personal Discrimination

Broadly consistent with other discrimination measures (Latrofa et al., 2009; Giamo et al., 2012) and building upon previous work (Jetten et al., 2001, 2013), we developed two items asking participants at both time points the extent they agreed with the items: “*I feel people look down on me because of my situation*” and “*People have discriminated against me because of my situation.*” Responses to the two items were measured on a 7-point Likert scale from *“Strongly disagree”* to *“Strongly agree,”* and the items were correlated at T1 (*r* = 0.75) and at T2 (*r* = 0.79).

#### Perceived Group-Based Discrimination

On the same 7-point Likert scale, perceived discrimination of homeless people as a group was also assessed. Two items were used, “*Homeless people as a group face discrimination*” and “*There is prejudice against homeless people.*” The two items were highly correlated at T1 (*r* = 0.81) and at T2 (*r* = 0.94).

#### Multiple Group Membership

Two items at T1 and two items at T2 measured multiple group membership since living at the Salvation Army to assess the extent to which people belong to multiple social groups. The items were adapted from a 2-item scale by Jetten et al. (2010) and a 4-item scale by Haslam et al. (2008) to be suitable for the specific population. At T1, participants were asked, *“Since coming to (name of Salvation Army Homeless Shelter), I am a member of lots of different social groups”* and *“Since coming to (name of Salvation Army Homeless Shelter), I have friends who are in lots of different groups.”* The two items were measured on a 7-point Likert scale with responses ranging from *“Do not agree at all”* to *“Agree,”* and highly inter-correlated (*r* = 0.66). On a similar 7-point scale, at T2 participants were asked, “*After living at (name of Salvation Army Homeless Shelter), I am a member of lots of different social groups,”* and “*After living at (name of Salvation Army Homeless Shelter), I have friends who are in lots of different groups.”* The two items were correlated (*r* = 0.78).

#### Personal Well-Being

Well-being was measured at T1 and T2. The personal well-being Index (PWI) developed by the International Well-being Group (2006) is an eight-item scale measuring satisfaction with life, covering eight quality of life domains (e.g., standard of living, achievement in life; personal relationships). An example item asks *“How satisfied are you with what you are currently achieving in life?”* (measured on a 10-point scale from “*completely dissatisfied*” to “*completely satisfied*”). The eight scores were averaged to give a score representing ‘subjective well-being,’ and for the purpose of comparing scores to Australian norms, the scores were standardized, so that each individual had a score between 0 and 100. The scale demonstrated good reliability (alpha at T1 = 0.84 and T2 = 0.94), and good validity (International Well-being Group, 2006).

## Results

### Path Analysis

The mean, SD and inter-correlations between measures of discrimination, multiple group membership and well-being are presented in **Table 1**. Consistent with the personal-group discrepancy (Postmes et al., 1999), it appeared that group-based discrimination was perceived to be higher than personal discrimination. In addition, at both time points, the number of groups that individuals belonged to was rated around the midpoint of the scale. The normative range on personal well-being in Australia is 73.4–76.4 points. The personal well-being in our sample was worse: our respondents were 10 points below this normative range. The average well-being score improved about 1 SD from T1 to T2.

**Table 1:** Mean and SD of discrimination measures, multiple group membership, and well-being.

		Personal discrimination		Group discrimination		Multiple group membership		Personal well-being	
	Mean (SD)	(T1)	(T2)	(T1)	(T2)	(T1)	(T2)	(T1)	(T2)
Perceived personal discrimination (T1) *(range: 1–7)*	3.83 (1.95)	1.00	0.57^∗∗^	0.43^∗∗^	0.37^∗∗^	-0.14	-0.28^∗^	-0.52^∗∗^	-0.35^∗∗^
Perceived personal discrimination (T2) *(range: 1–7)*	3.90 (1.97)		1.00	0.51^∗∗^	0.67^∗∗^	-0.07	-0.38^∗∗^	-0.47^∗∗^	-0.52^∗∗^
Perceived group-based discrimination (T1) *(range: 1–7)*	5.07 (1.93)			1.00	0.56^∗∗^	-0.17	-0.33^∗∗^	-0.20^∗^	-0.28^∗^
Perceived group-based discrimination (T2) *(range: 1–7)*	4.99 (1.98)				1.00	-0.02	-0.31^∗∗^	-0.27^∗^	-0.38^∗∗^
Multiple group membership since living at Salvation Army (T1) *(range: 1–7)*	3.54 (1.82)					1.00	0.39^∗∗^	0.35^∗∗^	0.17
Multiple group membership since living at Salvation Army (T2) *(range: 1–7)*	3.57 (1.89)						1.00	-0.34^∗∗^	0.43^∗∗^
Personal well-being (T1) *(range: 15.00–100)*	56.81 (18.40)							1.00	-0.57^∗∗^
Personal well-being (T2) *(range: 18.75–100)*	66.30 (19.31)								1.00

*p < 0.05, **p < 0.01, ***p < 0.001.

To assess the hypothesized relationships, we tested a structured model with measured variables using AMOS software (Version 22.0). Controlling for personal well-being at T1, we specified perceived personal discrimination (T1) and perceived group-discrimination (T1) as exogenous predictor variables. We specified multiple group membership (T2) as a mediator variable, with personal well-being (T2) as the outcome variable. To determine the fit of the model, we included several absolute and relative fit indices (see Hu and Bentler, 1999; Iacobucci, 2010), including the χ^2^ goodness-of-fit test, the comparative fit index (CFI), the root mean squared error of approximation (RMSEA), and Akaike’s Information Criterion (AIC).

**FIGURE 1:**
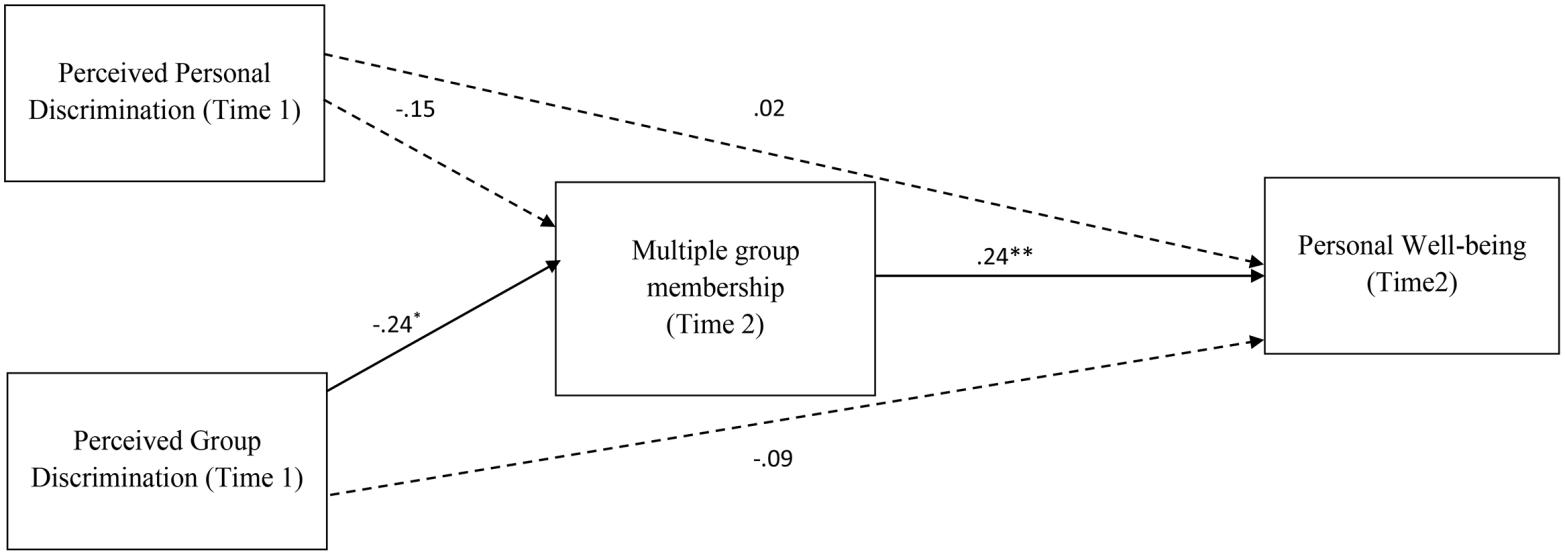
**Path model assessing the effects of perceived personal discrimination, perceived group discrimination on well-being (Time 2), through multiple group membership (Time 2).** Standardized parameter estimates shown. Dashed lines indicate non-significant relationships. ^∗^*p* = 0.056, ^∗∗^*p* < 0.05; ^+^Controlling for well-being at T1.

Our model fit the data well: χ^2^(1) = 5.11, *p* = 0.02, CFI = 0.96, RMSEA = 0.19, AIC = 43.11. **Figure 1** shows the standardized parameter estimates for the model. Group-based discrimination was negatively associated with gains in group membership at T2, which subsequently predicted well-being (T2). Perceived personal discrimination did not predict multiple group memberships and, although there were significant inter-correlations, in the model neither personal discrimination nor group-based discrimination directly predicted later well-being.

**FIGURE 2:**
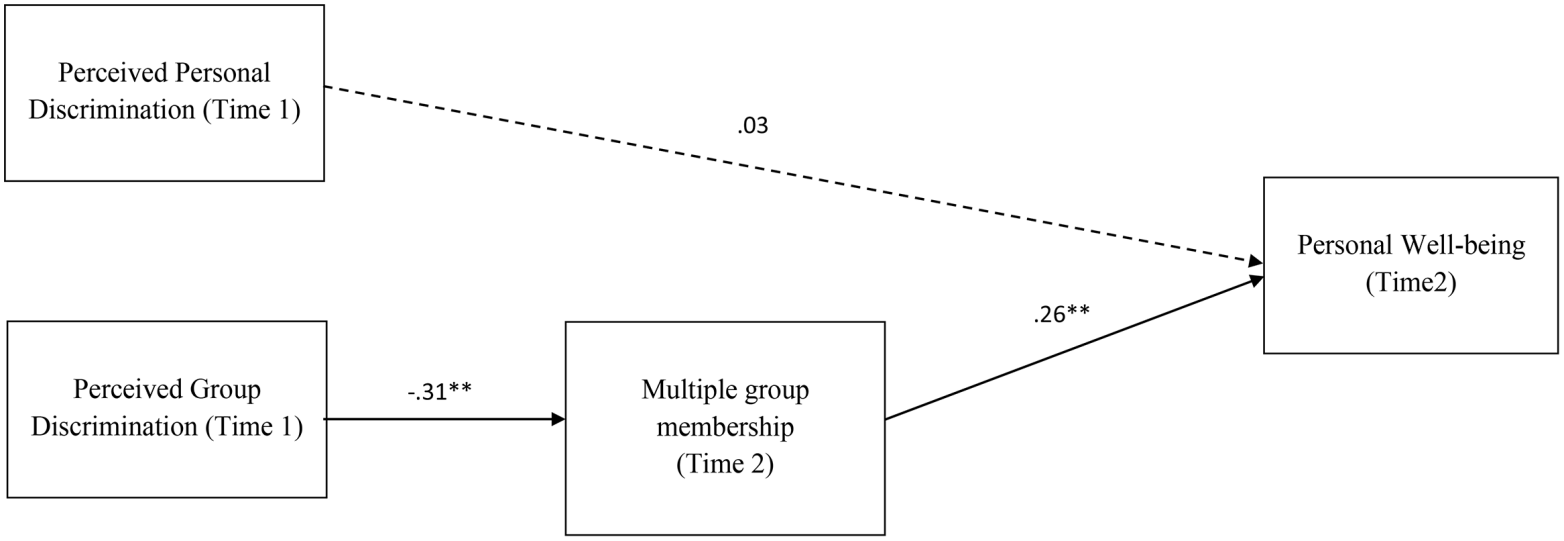
**Path model assessing the effects of perceived personal discrimination, perceived group discrimination on well-being (Time 2), through multiple group membership (Time 2).** Standardized parameter estimates shown. Dashed lines indicate non-significant relationships. ^∗^*p* < 0.05, ^∗∗^*p* < 0.01; ^+^Controlling for well-being at T1.

A refined model was tested, removing the pathway from personal discrimination to multiple group membership and the direct pathways from group-based discrimination to later well-being. This refined model showed an improved fit: χ^2^(3) = 7.12, *p* = 0.68, CFI = 0.95, RMSEA = 0.11, AIC = 41.12. **Figure 2** shows the standardized parameter estimates for the model. Again, group-based discrimination was significantly associated with fewer gains in group membership at T2, which subsequently predicted well-being (T2). Perceived personal discrimination did not predict later well-being.

### Alternative Model

**FIGURE 3:**
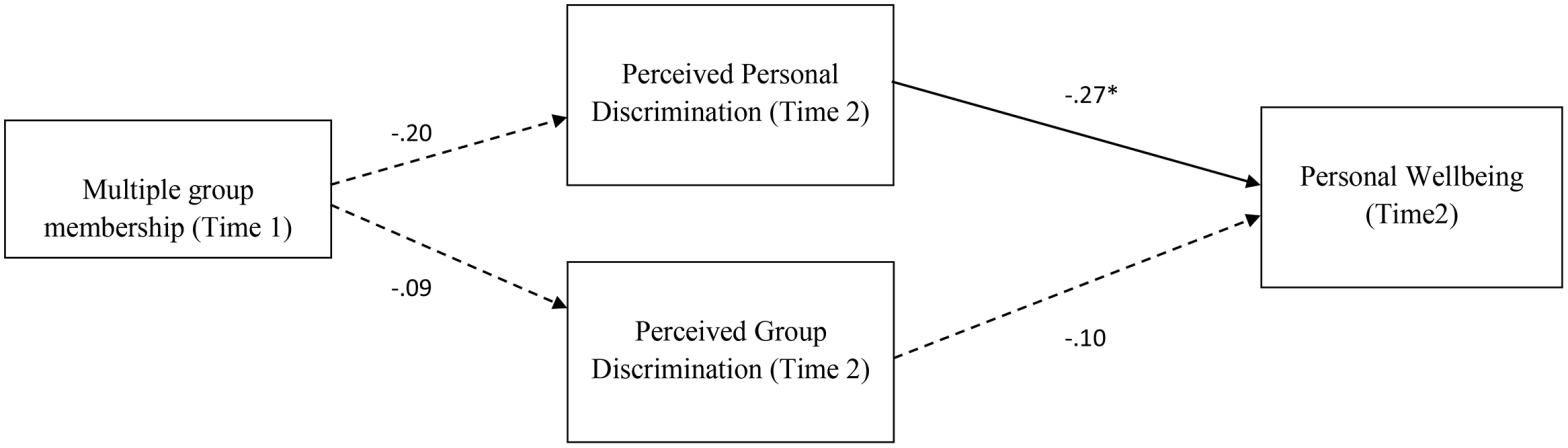
**Path model assessing the effects multiple group identities on perceived personal discrimination, perceived group discrimination and subsequent well-being (Time 2).** Standardized parameter estimates shown. Dashed lines indicate non-significant relationships. ^∗^*p* < 0.05, ^∗∗^*p* < 0.01; ^+^Controlling for well-being at T1.

In line with Major et al.’s (2002, 2003) argument that some people see discrimination more than others, it could be argued that those with multiple group membership perceive less discrimination. Put differently, being poorly connected and isolated means that one is more likely to see rejection. To test this alternative pathway, we specified a model where multiple group membership predicts discrimination perceptions. The alternative model did not fit the data as well as the previous models: χ^2^(2) = 61.55, *p* = 0.00, CFI = 0.39, RMSEA = 0.35, AIC = 93.55 (see **Figure 3**) and we retain the predicted models.

## Discussion

Although multiple group memberships can improve well-being, we predicted that homeless people who are arguably most in need of such identity resources are least likely to benefit from them because stigma and discrimination act as barriers against building social connections. Consistent with predictions, the experience of group-based discrimination was associated with fewer group memberships at T2. This suggests that group-based discrimination stands in the way of multiple group membership development at T2, and this negatively impacts on well-being. An alternative model where multiple group memberships at T1 predicts well-being at T2 through perceived personal and group-based discrimination did not fit the data as well as the hypothesized model.

Noteworthy too, perceived personal discrimination did not predict multiple group memberships. A possible explanation may be that discrimination directed at oneself does not affect seeking out of social connections and group memberships, whereas group-based discrimination does. Specifically, it is group-based discrimination that affects the salience of ‘us’ versus ‘them’ distinctions and self-categorisations. As our data suggest, this powerfully affects the orientation of the individual in the social world whereby belonging to a stigmatized group becomes a barrier for seeking out other groups (that are part of a more inclusive “we”) when facing discrimination on the basis of group membership.

Interestingly, when controlling for well-being at T1, there was no direct relationship between either measure of perceived discrimination and well-being at T2. There was only a relationship through multiple group membership, suggesting that for the homeless, this may be an important mechanism by which discrimination negatively affects health. That is, in particular for this population, the negative effects of discrimination may not so much be due to the painfulness of rejection, but more to the fact that group-based discrimination stands in the way of seeking support from other groups. It is being cut off from social identity resources to cope with discrimination that appears to negatively affect well-being over time.

### Implications and Future Research

This work informs understanding of the experiences of people who are homeless. Specifically, it demonstrates how discrimination against the homeless can negatively impact social connections, and subsequent well-being. This work contributes to a growing body of research on the effect of multiple group membership on health (e.g., Jetten et al., 2014). While consistent with the existing research that more multiple group memberships are associated with enhanced well-being, this research provides a better understanding of how multiple group memberships are impacted within a more vulnerable population. Specifically, multiple group memberships and/or attempts at developing new connections amongst the homeless are hampered by experiences of discrimination. The findings suggests that for people experiencing homelessness, group-based discrimination may deter individuals from seeking out of social connections and group memberships, whereas discrimination directed at oneself does not affect the orientation in the social world.

It remains an empirical question whether the processes observed in the present research are unique to the homeless. There are some reasons to suspect that there are some important differences between the homeless and other stigmatized minority groups (e.g., on the basis of gender, ethnicity or age). For instance, the motivation to turn to other groups — other than their own minority group — following group-based discrimination may be higher among homeless individuals than among members of other stigmatized groups. Given the heterogeneous people who experience homelessness and the lack of identification with the homeless group itself (Walter et al., under review), it may be the case that for these individuals in particular, other groups may become more important sources of social support. The inability to join other groups (and the resulting negative well-being consequences of this) may therefore be felt more by homeless individuals than by members from other stigmatized groups.

Another reason why these findings may be population specific relates to the high levels of exclusion that homeless individuals face. Indeed, there are not many stigmatized groups in today’s world that face this type of pervasive and legitimate discrimination (see Jetten et al., 2013 for a discussion). This would imply that the homeless are indeed a special case where the experience of discrimination may not necessarily lead to the provision and availability of support by others. The extent to which our findings can be generalized to other stigmatized groups should be examined in future research.

Even though the picture that is painted for the homeless looks bleak, these findings should not be taken as evidence that the homeless are powerless in the face of pervasive group-based discrimination. Indeed, there is now considerable evidence that suggest the contrary. For example, Johnson et al. (2008) demonstrated how people who were homeless actively managed and manipulated the stigma of homelessness to make sense of their worlds. In the context of outreach service provision, Parsell et al. (2014) demonstrated how people exercised agency and actively identified their sense of self and aspired trajectories to explain their exits from chronic homelessness. They demonstrated that people with experiences of homelessness were not passive service recipients whose housing status and identity was determined by the availability of social welfare and housing resources (Parsell et al., 2014).

There are limitations to this research that need noting. First, while our analyses meet the general rule of 10 cases per variable, our sample size was relatively small, reducing power. Further, whilst we control for initial levels of well-being, it should be noted that we did not control for other factors that could have affected the strength of relationships (e.g., mental illness, depression, psychosis, substance abuse). Having noted these weaknesses, our research also has a number of strengths. In particular, while we cannot assume causation, we did find that our associations were robust over a 3-months time period, in which participants were undergoing significant life changes and, for many, their situation at T2 was very different from T1.

Finally, these findings have important implications for those working with individuals who are homeless. Along with broader efforts to combat discrimination toward those experiencing homelessness, services structured to enhance group memberships where individuals are more integrated and connected, may enhance well-being and potentially contribute to breaking the cycle of homelessness. This is in line with other research findings showing group-based interventions for clients of homeless services provide well-being benefits beyond the stated purpose of the group activity. For instance, a study of homeless individuals attending a job- and life-skills program found that positive change in social network quality over time was associated with positive outcomes (specifically fewer individuals in the network using alcohol to intoxication; Gray et al., 2015). Our findings suggest that it may not be so much the building of social support networks, but the removal of barriers for people to turn to those social networks in times of need that is crucial in protecting the homeless’ long-term well-being.

## Conflict of Interest Statement

The authors declare that the research was conducted in the absence of any commercial or financial relationships that could be construed as a potential conflict of interest.

## Footnotes

1 Gender [completers vs. non-completers female: 59 vs. 42%, χ^2^(1, *N* = 119) = 3.32, *p* = 0.069], age [completers *M* = 34.3, SD = 9.05; non-completers *M* = 37.4, SD = 9.62, *F*(1,117) = 0.3.10, *p* = 0.081], employment status [completers vs. non-completers employed: 20% vs. 16%, χ^2^(1, *N* = 119) = 0.22, *p* = 0.64], alcohol consumption [completers *M* = 11.57, SD = 10.09; non-completers *M* = 15.08, SD = 10.09, *F*(1,92) = 2.64, *p* = 0.108],well-being [completers *M* = 5.68, SD = 1.79; non-completers *M* = 5.67, SD = 1.95, *F*(1,117) = 0.001, *p* = 0.981].
